# *Synthetic Biology*: fostering the cyber-biological revolution

**DOI:** 10.1093/synbio/ysw001

**Published:** 2016-05-27

**Authors:** Jean Peccoud

**Affiliations:** Editor-in-Chief

## Abstract

Since the description, in 2000, of two artificial gene networks, synthetic biology has emerged as a new engineering discipline that catalyzes a change of culture in the life sciences. Recombinant DNA can now be fabricated rather than cloned. Instead of focusing on the development of ad-hoc assembly strategies, molecular biologists can outsource the fabrication of synthetic DNA molecules to a network of DNA foundries. Model-driven product development cycles that clearly identify design, build, and test phases are becoming as common in the life sciences as they have been in other engineering fields. A movement of citizen scientists with roots in community labs throughout the world is trying to democratize genetic engineering. It challenges the life science establishment just like visionaries in the 70s advocated that computing should be personal at a time when access to computers was mostly the privilege of government scientists. Synthetic biology is a cultural revolution that will have far reaching implications for the biotechnology industry. The work of synthetic biologists today prefigures a new generation of cyber-biological systems that may to lead to the 5^th^ industrial revolution. By catering to the scientific publishing needs of all members of a diverse community, *Synthetic Biology* hopes to do its part to support the development of this new engineering discipline, catalyze the culture changes it calls for, and foster the development of a new industry far into the twenty first century.

On January 20, 2000, Nature published two articles reporting the design, fabrication, and characterization of two artificial gene networks. Timothy Gardner, Jim Collins, and Charles Cantor described a genetic toggle switch that could be flipped between an ON and OFF states using transient environmental signals [1]. Michael Elowitz and Stanislas Leibler described the Repressilator, a genetic circuit that exhibited oscillations of the expression of a reporter gene [2].

On the face of it, these two articles looked like biology papers. They included the description of new plasmids and reported data collected with instruments commonly used by biologists. And there was nothing particularly new in these experiments. Many molecular biologists had the skills necessary to assemble and characterize these plasmids but none of them thought of designing them. It took the minds of a mechanical engineer (T. Gardner) and a physicist (M. Elowitz) to imagine these circuits. The novelty of these articles was not so much in their biological aspect as it was in the applications of engineering principles to the design of circuits encoded in DNA molecules. These two articles have been a source of inspiration for many of us. They have catalyzed the emergence of a movement of dreamers aspiring to engineer DNA like their parents engineered silicon. This movement eventually led to the emergence of synthetic biology as a new field of engineering [3–5].

Fifteen years later, we have come to appreciate the culture change that synthetic biology calls for. We see many indications that this specialty has the potential to support an industrial revolution fueled by the emergence of cyber-biological systems across many segments of the economy. The dynamics between scientific breakthroughs and innovative industrial applications is well illustrated by the career paths of the discipline pioneers. Gardner left academia for industry 10 years ago to join one of the first synthetic biology startups while Elowitz stayed in academia where his work continues to deeply renew our understanding of biological processes.

## DNA is the new silicon

In the early days of genetic engineering, techniques used to assemble recombinant DNA molecules were extremely limited. They extensively relied on the presence of motifs that could be cut by restriction enzymes. The resulting restriction fragments could be separated by physical purification processes like electrophoresis and stitched together using DNA ligases. In the 80s, the availability of instruments automating the synthesis of small single-stranded DNA molecules called oligonucleotides along with the invention of the Polymerase Chain Reaction (PCR) [6, 7] led to the development of site-directed mutagenesis strategies. It became possible to locally edit natural DNA sequences by introducing new restriction sites, eliminating others, and altering the biological functions of specific DNA sequences. However, this process was expensive, time-consuming, and very constrained by the specific characteristics of individual DNA sequences. As a result, biologists have been concerned by the challenges of assembling new plasmids from existing genetic material since the early days of genetic engineering [8]. A number of new techniques have made it easier to recombine DNA fragments but little has changed in the way most biologists approach the development of new DNA molecules to express their genes of interest. Their attention is disproportionately focused on the assembly of expression vectors at the expense of other aspects of their research project [9]. Many research and development projects in academia and industry are constrained by the perceived cost and limitations of producing new recombinant DNA molecules.

One of the most transformative ideas introduced by synthetic biology pioneers like Drew Endy and Tom Knight [3] is that DNA should be “fabricated” instead of being handcrafted. Fabrication and manufacturing are words that imply that DNA molecules should be the output of industrial processes instead of the artisanal production of skilled craftsmen. The vision of an industrial production of new DNA molecules calls for generic assembly processes that can be applied to any DNA sequence instead of the *ad-hoc* cloning processes commonly used by molecular biologists [10]. It also anticipates the emergence of high-throughput assembly lines depending on automated instruments and factory workers, who may not need advanced degrees to perform the tasks that cannot be automated [11]. This evolution is reminiscent of the evolution of oligonucleotide synthesis, which is now mostly outsourced to a few companies like Integrated DNA Technologies.

Early on, synthetic biologists have anticipated the emergence of a DNA synthesis industry providing custom fabrication services to the biotechnology industry comparable to the foundries serving the semi-conductor industry. DNA synthesis, also known as “gene synthesis”, is the *de novo* synthesis of DNA molecules entirely derived from oligonucleotides produced using a chemical process [12]. DNA synthesis is not new. It is as old as molecular biology itself as it was instrumental in the elucidation of the genetic code in the 60s [13–15]. However, it is only in the 90s that oligonucleotides became cheap enough to make it affordable to order the large numbers needed for DNA synthesis projects. At the same time, the rapid development of the PCR provided enzymes and protocols that could be used to assemble many oligonucleotides in a single reaction with the fidelity needed to meet the quality requirements of *de novo* DNA synthesis [16]. Around 2000, a number of startups like Blue Heron, GeneArt, and DNA2.0 launched gene synthesis services using techniques developed in the 90s. Many established companies serving the life science industry followed suit by offering DNA synthesis in addition to other services like oligonucleotide synthesis (Integrated DNA Technologies) or DNA sequencing (Genewiz). The cost of DNA synthesis services has steadily decreased but it is still regarded as prohibitively expensive for many projects. As a result, most projects still heavily rely on traditional cloning techniques and limit the use of DNA synthesis to the generation of specific sequences like codon-optimized open reading frames. This limited use of DNA synthesis has motivated the emergence of a second generation of DNA synthesis companies (Twist Biosciences, Gen9, SGI-DNA) hoping to disrupt the DNA synthesis market by developing new synthesis technologies that will reduce the cost, increase the throughput, and reduce times of DNA synthesis by orders of magnitude.

## Model-driven development lifecycle

**Figure 1.**
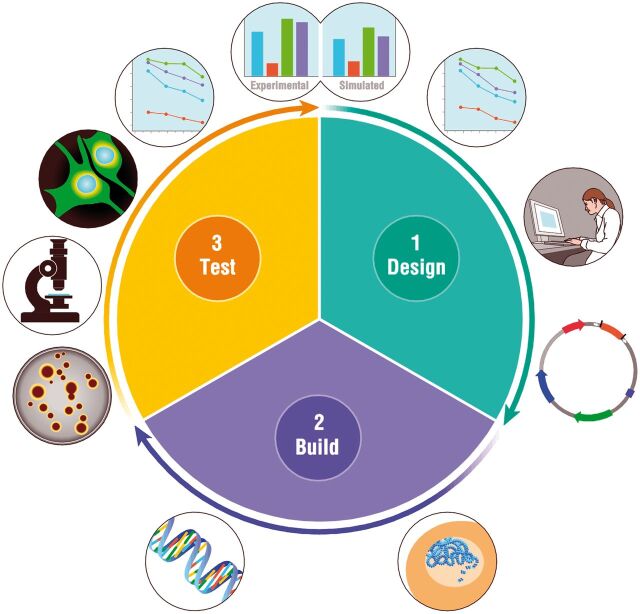
Product development lifecycle: Like devices produced by other industries, synthetic biology products are developed through several iterations of a design-build-test cycle. In the design phase, computer models are used to generate DNA sequences and predict their properties. In the build phase, these DNA molecules are produced by manufacturing processes that assemble large DNA molecules out of chemically synthesized building blocks. Finally, in the testing phase, DNA is introduced in living cells and gene expression is measured. Experimental data is finally compared to simulation results to improve the design in the next iteration of this cycle.

Another benefit of using the word “fabrication” in relation to DNA is that it implicitly refers to the life cycle of a product (Figure 1). It places the assembly of synthetic DNA molecules in relation to other stages upstream and downstream of fabrication. When biologists are freed from the hassle of making DNA molecules, they can allocate more resources to imagining the DNA sequences that best serve their research objectives. Considering that fabrication is orthogonal to the design of DNA molecules can unleash the creativity of life scientists. Instead of being constrained by the limits of what DNA molecules they could write, they can now think of what DNA molecules they should write and let someone else figure out how to make them. While ideally fabrication should be independent of design, in practice it is not. Vendors and methods have restrictions around certain sequences (high GC content, sequence repeats, motifs). In practice this clean separation does not yet exist and genetic designers need to be mindful of the manufacturability of their designs. 

**Figure 2.**
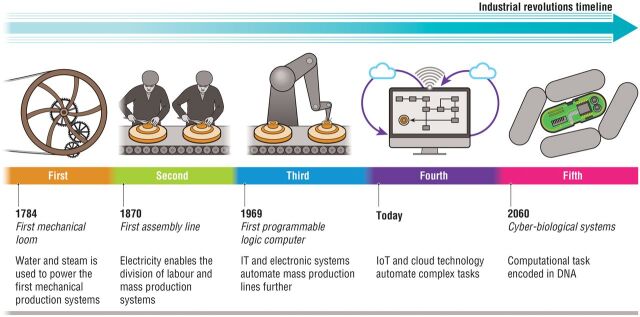
Fifth industrial revolution: Synthetic biology is developing a new generation of cyber-biological systems that have the potential to catalyze the 5^th^ industrial revolution in the second half of the 21^st^ century.

The benefits of *de novo* gene synthesis cannot be understated. It has made it possible to “resurrect” extinct viral strains [17], rationally attenuate viral genomes [18, 19], refactor the genomes of bacteria [20] and yeast [21], and extend the genetic code [22]. These moonshot projects drew a lot of attention but they pale in comparison of the upcoming transformations of the biotechnology supply chain resulting from cheap DNA synthesis. One can anticipate that in a not-too-distant future it may become cheaper to resynthesize DNA molecules from scratch rather than storing and distributing existing plasmids. For the sake of argument, imagine that gene synthesis rates reach the symbolic threshold of 1 penny per base pair. A 5kb plasmid could be synthesized for $50, less than the price of ordering it from a not-for-profit organization like Addgene [23]. At that rate, the biological sample that contains the DNA molecules becomes much less valuable than the information about the DNA sequence itself. Beyond the cost factor, the time to access samples is very important. In the foreseeable future, retrieving an existing sample from a freezer will be much faster than synthesizing it and could justify the biobanking expenses.

Upstream of fabrication, the design of DNA molecules is now often model-driven. Optimization of coding sequences to maximize expression of heterologous proteins often referred to as “codon-optimization” is one of the most popular forms of computational design of synthetic DNA sequences [24–27]. The rational attenuation of viral sequences [18, 19, 28] and the computation of ribosome binding sites are other examples of model-driven design of DNA [29]. A number of other computational methods are being developed to streamline the design of longer and more complex DNA sequences [30–33] but the predictive power of mathematical models of the behaviors encoded in DNA sequences is still limited. The development of gene networks that implement user-defined specifications still requires a lot of empirical tuning to achieve the desired phenotypes [31]. This observation feeds a debate among synthetic biologists about the respective roles of rational design and evolutionary methods [34].

Downstream of fabrication, the phenotypes encoded in synthetic DNA molecules are now analyzed using quantitative models. When gene expression was measured by looking at bands on electrophoresis gels, data analysis was limited to qualitative (the protein is present or absent) or semi-quantitative (the protein is highly expressed) statements. The development of fluorescent proteins that could be used as reporter genes has opened numerous possibilities of more rigorous mathematical analysis [35–39]. Fluorescent proteins made it possible to measure gene expression in live cells instead of having to measure proteins extracted from cell cultures. Fluorescent proteins also made it possible to collect data with a single cell resolution using commonly available instruments like microscopes and flow-cytometers. Despite well-known limitations, the use of fluorescent proteins as reporter genes provided the data needed to develop sophisticated mathematical models of gene expression [40]. More recently, the integration of imaging and microfluidics [41–44] has greatly improved the quality of data available to modelers.

## “The Times They Are a-Changin”

By using recombinant DNA technologies in the context of a broader model-driven product development workflow, synthetic biology has finally brought “engineering” to “genetic engineering”. This represents a major culture change that has been triggered by a new interest of engineers and quantitative scientists for DNA. This demographic trend is just beginning and will take a few decades to complete. For the most part, the generation of scientists trained as biologists will not be able to embrace this change in their lifetime. It will take a generation of young biological engineers with solid quantitative and computational skills for the biotechnology industry to complete the transition. For more than 10 years, the competition iGEM has communicated to thousands of undergraduate students an inspiring vision of a world where DNA should be simple to engineer [45, 46]. Over the course of a summer, students often come to appreciate the gap between this compelling vision and today’s reality. Yet, their dream lives on and will motivate them to spearhead the culture changes that will transform the biotechnology industry.

The emergence of the DIYBio and citizen scientist movements also participates in this culture change in the sense that DIYBiologists are taking some research projects away from the biological establishment [47–50]. DIYBiologists have the ambition to democratize biological research by bringing it to their kitchen and their garage just like the personal computing movement in the 70s challenged the dominance of government computing infrastructures. They are excited to challenge the incumbents who dominate the biotechnology industry. The Open Insulin Project is a good illustration of this trend. However, most DIYbiologists are hobbyists working on projects of very limited scope. Even initiatives with catchy names that manage to generate a short-lived hype are most likely to be soon forgotten. However, the development of Ginkgo Bioworks is evocative of Silicon Valley mythology. It didn’t start in a garage but in a repurposed shipping container [50], which is close enough to fuel the imagination of aspiring entrepreneurs. Ten years, many federal grants and contracts, and several rounds of funding later, Ginkgo is sustaining its growth by attracting world class talent with its very distinctive corporate culture that comes with strong flavors of biohacking.

## The cyber-biological industrial revolution

This culture change has the potential to enable an industrial revolution. Recently the world economic forum has recognized the emergence of cyber-physical systems as catalysts of the 4^th^ industrial revolution [51]. Cyber-physical systems are hybrid systems composed of a number of physical entities connected to software running control algorithms to direct individual devices in response to data received from various feeds. The navigation apps running on mobile devices (Google Maps, Waze) create a cyber-physical transportation system that many people use on a daily basis. Smart phones provide position and traffic information to a central server. The information provided by this network of devices is analyzed in real-time and along with other data sources to provide individualized directions to each user. Beyond transportation, the power grid, manufacturing, retail, health-care, and air-traffic control now include many cyber-physical systems.

With its strong emphasis on model-driven biology, synthetic biology also includes cyber-physical systems. For instance, the manufacturing of custom DNA molecules is a physical process driven by several layers of software. Virtual labs like Transcriptic or Emerald Cloud Labs are also examples of cyber-physical systems in biotechnology. However, synthetic biology goes beyond this by encoding control algorithms within DNA molecules, engineering organisms that can reproduce, communicate with each other, or leverage complex webs of interactions between hosts and pathogens, preys and predators, etc. There is an unprecedented level of complexity in these engineered biological systems that makes them different from cyber-physical systems. They may be best described as “cyber-biological” (Figure 2).

Synthetic biology is certainly not as mature as the technologies that catalyzed the emergence of cyber-physical systems. It is still mostly very artisanal but there are early indications that cyber-biological systems have the potential to catalyze the fifth industrial revolution in the second half of the twenty-first century. It is important to remember that the Internet and the Global Positioning Systems, two key technologies that enabled the development of today's cyber-physical systems, were developed by the US Department of Defense more than forty years ago [52]. This historical perspective helps one to appreciate the significance of the investments that the Defense Advanced Research Project Agency (DARPA) has been making in synthetic biology over the last few years. Its “Living Foundries” [53] program articulated a vision of a new industry relying on cyber-biological systems. This frontier is so important to DARPA that they recently created a new office of Biological Technologies [54].

There is also evidence that the center of gravity of the synthetic biology community has been progressively shifting toward industry. Companies like Amyris, Synthetic Genomics, Gingko Bioworks, Intrexon, or Twist Biosciences have raised resources that allow them to develop industrial-scale research infrastructures beyond the reach of academic research groups. SynBERC's very successful industry program has inspired some mature companies to develop synthetic biology initiatives in house or through collaborations with synthetic biology startups.

## Editorial Policies

**Figure 3.**
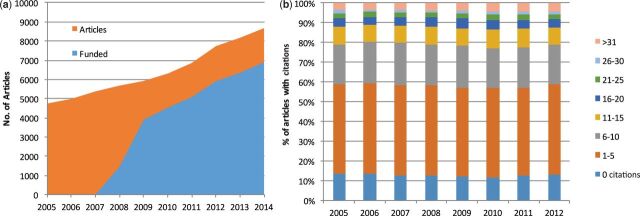
Bibliometric analysis of synthetic biology (A) The number of synthetic biology papers published has been increasing at 6% per year between 2005 and 2014. It is expected that this trend will continue as the vast majority of these articles now cite one or more grants or contracts supporting the work. (B) The field of synthetic biology shows a strong citation patterns as 40% of papers receive more than 5 citations in the first two years after publication.

Synthetic biology articles are still mostly published in a broad range of interdisciplinary and specialized journals [55]. This situation can be problematic as it makes it difficult for readers to identify relevant papers and for authors to get published. In this environment *Synthetic Biology* aims to be a common forum in which researchers can share research and ideas. The number of synthetic biology articles has been growing at an annual rate of 6% over the last 10 years and this rate is expected to be sustained over the next ten years as governments across the globe have been investing in synthetic biology programs (Figure 3A). The citation behavior of the field is very strong, with only 13% of 2012 papers receiving 0 citations and 12% receiving 16+ citations. From 2005, each year over 40% of papers were cited more than 5 times in their first two years. This indicates a history of strong citation performance in the field (Figure 3B). 

It is expected that a journal dedicated to the field will increase the visibility of synthetic biology papers, which will translate in improved citation statistics. Yet, *Synthetic Biology* refrains from making editorial decisions based on the editors’ assessment of the anticipated impact of the work presented in the submissions it receives. The journal publishes all articles that are scientifically sound as evaluated by a rigorous peer-review process.

In order to minimize reproducibility issues, authors are requested to provide comprehensive sets of supporting data in a computer readable format. *Synthetic Biology* encourages authors to use data repositories like Figshare or Dryad to deposit their data prior to submitting their manuscript in a format allowing reviewers and readers to reuse the data. DNA sequences are of particular importance to most synthetic biology articles and authors will be requested to provide the complete sequences of the plasmids and other genetic material described in the manuscript [56]. Authors are also encouraged to release both raw data generated by instruments and the reduced data sets presented in the articles figures. For example, papers using time lapse microscopy to measure the dynamics of gene expression should release the images produced by the microscope (raw data), the scripts used to extract gene expression data from series of images, and the gene expression data (reduced data) used to generate figures [57–59]. The use of commonly accepted file formats and community standards like SBML [60] and SBOL [61, 62] is recommended. For example, DNA sequences can be deposited as fasta, genbank, or SBOL files but PDF files are not suitable to communicate DNA sequences.

Reviewers are attentive to the proper use of physical units and calibration methods to ensure the reproducibility of results reported in the journal. It is common for fluorescence data to be reported as relative units making it impossible to compare datasets. Calibration of the instrument can lead to higher quality data [63].

*Synthetic Biology* recognizes the important contributions of industry to the development of the field. Manuscripts based on commercially available resources such as strains, reagents, or software are welcome as long as authors fully disclose their conflict of interest. Authors reporting results produced with their company products should keep in mind that their submissions need to meet the journal rigorous scientific standards and successfully go through peer-review. Submissions that read like promotional material are rejected without review.

The journal encourages the release of computational resources using one of the Open Source licenses recognized by the Open Source Initiative. However, we also recognize that in some instances, open source release may jeopardize the long term sustainability of important computing resources. Therefore, open source release is not a requirement to the publication of articles describing new software, databases, or web sites.

*Synthetic Biology* acknowledges that engineers and life scientists have different publishing usages. Engineers and physicists commonly rely on preprint servers and conferences to disseminate scientific results. *Synthetic Biology* supports these traditions that provide quick access to new scientific results while providing a new avenue to publish these results in a more polished format that readers will identify and cite more easily.

Finally, *Synthetic Biology* is interested in receiving submissions from students and teachers reporting educational projects. Submissions from citizen scientists discussing topics of interest to the DIYBio community are welcome. Authors of educational and DYIBio papers should send a pre-submission enquiry to the editorial office (synbio.editorialoffice@oup.com) in order to ensure that their ideas are a good fit for the journal.

By catering to the scientific publishing needs of all members of a diverse community, *Synthetic Biology* hopes to do its part to support the maturation of this new engineering discipline, catalyze the culture changes it calls for, and prepare for the emergence of a new industry far into the twenty first century.

## References

[1] Gardner T.S. Cantor C.R. Collins J.J., Construction of a genetic toggle switch in Escherichia coli. Nature, 2000 403(6767): p. 339–42. 10659857 10.1038/35002131

[2] Elowitz M.B. Leibler S., A synthetic oscillatory network of transcriptional regulators. Nature, 2000 403(6767): p. 335–338. 10659856 10.1038/35002125

[3] Endy D., Foundations for engineering biology. Nature, 2005 438(7067): p. 449–53. 16306983 10.1038/nature04342

[4] Baker D., , Engineering life: building a fab for biology. Sci.Am., 2006 294(6): p. 44–51. 10.1038/scientificamerican0606-44 16711359

[5] Benner S.A. Sismour A.M., Synthetic biology. Nature Reviews Genetics, 2005 6(7): p. 533–543. 10.1038/nrg1637 PMC7097405 15995697

[6] Saiki R.K., , Primer-directed enzymatic amplification of DNA with a thermostable DNA polymerase. Science, 1988 239: p. 487–91. 2448875 10.1126/science.2448875

[7] Saiki R.K., , *Enzymatic amplification of beta-globin genomic sequences and restriction site analysis for diagnosis of sickle cell anemia*. Science. Science, 1985 230: p. 1350–4. 2999980 10.1126/science.2999980

[8] Sambrook J. Russell D.W., Molecular cloning : a laboratory manual. 3rd ed. 2001, Cold Spring Harbor, N.Y: Cold Spring Harbor Laboratory Press.

[9] Peccoud J., Cloning forever. The Winnower, 2015 3: p. e143018.86995

[10] Ellis T. Adie T. Baldwin G.S., DNA assembly for synthetic biology: from parts to pathways and beyond. Integrative Biology, 2011 3(2): p. 109–18. 21246151 10.1039/c0ib00070a

[11] Notka F. Liss M. Wagner R., Industrial scale gene synthesis. Methods Enzymol, 2011 498: p. 247–75. 21601681 10.1016/B978-0-12-385120-8.00011-5

[12] Czar M.J., , Gene synthesis demystified. Trends Biotechnol, 2009 27(2): p. 63–72. 19111926 10.1016/j.tibtech.2008.10.007

[13] Khorana H.G., , Polynucleotide synthesis and the genetic code. Cold Spring Harb Symp Quant Biol, 1966 31: p. 39–49. 5237635 10.1101/sqb.1966.031.01.010

[14] Khorana H.G., Synthetic nucleic acids and the genetic code. JAMA, 1968 206(9): p. 1978–82. 5754917

[15] Agarwal K.L., , Total synthesis of the gene for an alanine transfer ribonucleic acid from yeast. Nature, 1970 227(5253): p. 27–34. 5422620 10.1038/227027a0

[16] Stemmer W.P., , Single-step assembly of a gene and entire plasmid from large numbers of oligodeoxyribonucleotides. Gene, 1995 164(1): p. 49–53. 7590320 10.1016/0378-1119(95)00511-4

[17] Tumpey T.M., , Characterization of the reconstructed 1918 Spanish influenza pandemic virus. Science, 2005 310(5745): p. 77–80. 16210530 10.1126/science.1119392

[18] Mueller S., , Live attenuated influenza virus vaccines by computer-aided rational design. Nature Biotechnology, 2010 28(7): p. 723–U1729. 10.1038/nbt.1636 PMC2902615 20543832

[19] Coleman J.R., , Virus attenuation by genome-scale changes in codon pair bias. Science, 2008 320(5884): p. 1784–7. 18583614 10.1126/science.1155761 PMC2754401

[20] Gibson D.G., , Creation of a bacterial cell controlled by a chemically synthesized genome. Science, 2010 329(5987): p. 52–6. 20488990 10.1126/science.1190719

[21] Annaluru N., , Total synthesis of a functional designer eukaryotic chromosome. Science, 2014 344(6179): p. 55–8. 24674868 10.1126/science.1249252 PMC4033833

[22] Chin J.W., Expanding and reprogramming the genetic code of cells and animals. Annu Rev Biochem, 2014 83: p. 379–408. 24555827 10.1146/annurev-biochem-060713-035737

[23] Kamens J., The Addgene repository: an international nonprofit plasmid and data resource. Nucleic Acids Res, 2014. in press. 10.1093/nar/gku893 PMC4384007 25392412

[24] Papamichail D, , Codon Context Optimization in Synthetic Gene Design. IEEE/ACM Trans Comput Biol Bioinform, 2016. 10.1109/TCBB.2016.2542808 27019501

[25] Boel G., , Codon influence on protein expression in E. coli correlates with mRNA levels. Nature, 2016 529(7586): p. 358–63. 26760206 10.1038/nature16509 PMC5054687

[26] Chin J.X. Chung B.K. Lee D.Y., Codon Optimization OnLine (COOL): a web-based multi-objective optimization platform for synthetic gene design. Bioinformatics, 2014 30(15): p. 2210–2. 24728853 10.1093/bioinformatics/btu192

[27] Gustafsson C. Govindarajan S. Minshull J., Codon bias and heterologous protein expression. Trends Biotechnol, 2004 22(7): p. 346–53. 15245907 10.1016/j.tibtech.2004.04.006

[28] Fan R.L., , Generation of Live Attenuated Influenza Virus by Using Codon Usage Bias. J Virol, 2015 89(21): p. 10762–73. 26269186 10.1128/JVI.01443-15 PMC4621104

[29] Salis H.M. Mirsky E.A. Voigt C.A., Automated design of synthetic ribosome binding sites to control protein expression. Nat Biotechnol, 2009 27(10): p. 946–50. 19801975 10.1038/nbt.1568 PMC2782888

[30] Nielsen A.A., , Genetic circuit design automation. Science, 2016 352(6281): p. aac7341. 27034378 10.1126/science.aac7341

[31] Lux M.W., , Genetic design automation: engineering fantasy or scientific renewal? Trends in Biotechnology, 2012 30(2): p. 120–126. 22001068 10.1016/j.tibtech.2011.09.001 PMC3779889

[32] Myers C.J, . Genetic design automation. in ICCAD '09 Proceedings of the 2009 International Conference on Computer-Aided Design. 2009. ACM.

[33] Densmore D.M. Bhatia S., Bio-design automation: software + biology + robots. Trends Biotechnol, 2014 32(3): p. 111–3. 24269087 10.1016/j.tibtech.2013.10.005

[34] Silver P.A., , Synthetic biology: Engineering explored. Nature, 2014 509(7499): p. 166–7. 24805338 10.1038/509166a

[35] Bintu L., , Dynamics of epigenetic regulation at the single-cell level. Science, 2016 351(6274): p. 720–4. 26912859 10.1126/science.aab2956 PMC5108652

[36] Lin Y., , Combinatorial gene regulation by modulation of relative pulse timing. Nature, 2015 527(7576): p. 54–8. 26466562 10.1038/nature15710 PMC4870307

[37] Uphoff S., , Stochastic activation of a DNA damage response causes cell-to-cell mutation rate variation. Science, 2016 351(6277): p. 1094–7. 26941321 10.1126/science.aac9786 PMC4827329

[38] Huh D. Paulsson J., Non-genetic heterogeneity from stochastic partitioning at cell division. Nat Genet, 2011 43(2): p. 95–100. 21186354 10.1038/ng.729 PMC3208402

[39] Lestas I. Vinnicombe G. Paulsson J., Fundamental limits on the suppression of molecular fluctuations. Nature, 2010 467(7312): p. 174–8. 20829788 10.1038/nature09333 PMC2996232

[40] Young J.W., , Measuring single-cell gene expression dynamics in bacteria using fluorescence time-lapse microscopy. Nat Protoc, 2012 7(1): p. 80–8. 10.1038/nprot.2011.432 PMC4161363 22179594

[41] Linshiz G., , End-to-end automated microfluidic platform for synthetic biology: from design to functional analysis. J Biol Eng, 2016 10: p. 3. 26839585 10.1186/s13036-016-0024-5 PMC4736182

[42] Shih S.C., , A Versatile Microfluidic Device for Automating Synthetic Biology. ACS Synth Biol, 2015 4(10): p. 1151–64. 26075958 10.1021/acssynbio.5b00062

[43] Ball D.A., , Adaptive imaging cytometry to estimate parameters of gene networks models in systems and synthetic biology. PLoS One, 2014 9(9): p. e107087. 25210731 10.1371/journal.pone.0107087 PMC4161401

[44] Ferry M.S. Razinkov I.A. Hasty J., Microfluidics for synthetic biology: from design to execution. Methods Enzymol, 2011 497: p. 295–372. 21601093 10.1016/B978-0-12-385075-1.00014-7 PMC4836382

[45] Goodman C., Engineering ingenuity at iGEM. Nature Chemical Biology, 2008 4(1): p. 13. 18084272 10.1038/nchembio0108-13

[46] Smolke C.D., Building outside of the box: iGEM and the BioBricks Foundation. Nature Biotechnology, 2009 27(12): p. 1099–1102. 10.1038/nbt1209-1099 20010584

[47] Kuiken T., DIYbio: Low Risk, High Potential. Scientist, 2013 27(3): p. 26–27.

[48] Delgado A., DIYbio: Making things and making futures. Futures, 2013 48: p. 65–73.

[49] *Empowering citizen scientists*. *Scientists should consider engaging more with the DIYbio community*. Nat Methods, 2015 12(9): p. 795. 26554083

[50] Alper J., Biotech in the basement. Nature Biotechnology, 2009 27(12): p. 1077–1078. 10.1038/nbt1209-1077 20010575

[51] Maynard A.D., Navigating the fourth industrial revolution. Nat Nanotechnol, 2015 10(12): p. 1005–6. 26632281 10.1038/nnano.2015.286

[52] Jacobsen A., The Pentagon's brain : an uncensored history of DARPA, America's top secret military research agency. First edition. ed. 2015, New York, NY: Little, Brown and Company. viii, 552 pages, 16 unnumbered pages of plates.

[53] Pennisi E., Synthetic biology. DARPA offers $30 million to jump-start cellular factories. Science, 2011 333(6039): p. 147. 21737716 10.1126/science.333.6039.147

[54] Laursen L., DARPA redesign. Nat Biotech, 2014 32(6): p. 509–509.

[55] Peccoud J. Isalan M., The PLOS ONE Synthetic Biology Collection: Six Years and Counting. Plos One, 2012 7(8): p. 7. 10.1371/journal.pone.0043231 PMC3419720 22916228

[56] Peccoud J., , Essential information for synthetic DNA sequences. Nature Biotechnology, 2011 29(1): p. 22–22. 10.1038/nbt.1753 21221092

[57] Ioannidis J.P.A., How to Make More Published Research True. Plos Medicine, 2014 11(10). 10.1371/journal.pmed.1001747 PMC4204808 25334033

[58] Nosek B.A., , SCIENTIFIC STANDARDS. Promoting an open research culture. Science, 2015 348(6242): p. 1422–5. 26113702 10.1126/science.aab2374 PMC4550299

[59] Wilson G., , Best practices for scientific computing. PLoS Biol, 2014 12(1): p. e1001745. 24415924 10.1371/journal.pbio.1001745 PMC3886731

[60] Hucka M., , The systems biology markup language (SBML): a medium for representation and exchange of biochemical network models. Bioinformatics, 2003 19(4): p. 524–31. 12611808 10.1093/bioinformatics/btg015

[61] Quinn J.Y., , SBOL Visual: A Graphical Language for Genetic Designs. PLoS Biol, 2015 13(12): p. e1002310. 26633141 10.1371/journal.pbio.1002310 PMC4669170

[62] Galdzicki M., , The Synthetic Biology Open Language (SBOL) provides a community standard for communicating designs in synthetic biology. Nat Biotechnol, 2014 32(6): p. 545–50. 24911500 10.1038/nbt.2891

[63] Beal J., Bridging the gap: a roadmap to breaking the biological design barrier. Front Bioeng Biotechnol, 2014 2: p. 87. 25654077 10.3389/fbioe.2014.00087 PMC4299508

